# The Buckling Behavior and Reliability Evaluation of a Cable-Stayed Bridge with Unique-Shaped Towers

**DOI:** 10.3390/ma17246124

**Published:** 2024-12-14

**Authors:** Yaoxiang Jia, Rujin Ma, Xiaoyu Zhou, Benjin Wang

**Affiliations:** 1 School of Aerospace Engineering and Applied Mechanics, Tongji University, Shanghai 200092, China; 2230870@tongji.edu.cn; 2 Department of Bridge Engineering, Tongji University, Shanghai 200092, China; rjma@tongji.edu.cn; 3 Shanghai Urban Construction Design and Research Institute (Group) Co., Ltd., Shanghai 200011, China; xyzhoutj@163.com

**Keywords:** cable-stayed bridge, unique-shaped tower, nonlinear buckling analysis, Monte Carlo simulation, reliability evaluation

## Abstract

Buckling is a significant concern for cable-stayed bridges that incorporate a large number of steel components, particularly those featuring unique-shaped towers that require further examination due to the intricate internal force and stress distribution. This paper investigates the buckling behavior of a cable-stayed bridge with inverted V-shaped towers. The cable tower is characterized by its unique design that consists of diagonal bracings and columns in a compression-bending state. A finite element model is established for the nonlinear buckling analysis of the bridge, revealing that the buckling failure mode of the bridge mainly concerns the tower columns that bear large bending moments and axial compressions. The buckling safety factors are analyzed under different loading conditions and design parameters, including the stiffening rib thickness, the width-to-thickness ratio, and the initial cable forces. It indicates that the design optimization can be achieved by using smaller and thinner ribs while maintaining the buckling safety factor above the required level in design specifications. Furthermore, the reliability evaluation of buckling safety is considered using Monte Carlo simulations, which incorporates the long-term effects of corrosion on steel components. Based on the identified buckling failure modes and safety factors, it suggests that the buckling resistance of the bridge is sufficient, though it can be further enhanced by using high-strength weathering steel on critical parts. Additionally, maintenance interventions are shown to be highly beneficial in improving the life-cycle performance of the structure.

## 1. Introduction

Cable-stayed bridges with unique-shaped towers have gained significant attention due to their distinctive design concepts and elements that contribute to the aesthetic values of bridge engineering projects. The non-traditional shape or configuration of the tower may introduce challenges in various aspects. In particular, the design of a unique-shaped tower may consist of a large number of steel members, for which buckling is a major concern in design standards [1,2].

In the design phase for compressed components, the linear buckling analysis, namely the eigenvalue buckling analysis, can be applied, and the corresponding design requirements and verification methods are provided for different categories of components based on yield strength and thickness-to-width ratio. In that case, the application of high-strength steel, e.g., Q420 to Q690, can significantly improve the buckling resistance, the enhanced strength of which allows HSS to resist deformation and buckling more effectively when subjected to the same loads [3,4,5]. Yet, it is incapable of taking into account the nonlinear behaviors of structural geometries and materials, and nonlinear buckling analyses are carried out for specific bridges [6,7,8,9,10]. Meanwhile, improved methods for buckling analyses are also proposed. A modified eigenvalue analysis on cable-stayed bridges is conducted for a member-based buckling concept, which introduces a fictitious axial force on the girder [11,12]. In addition, the convergence criteria for inelastic buckling were also proposed, which can consider the effect of the bending moments in steel frames. Similarly, an innovative continuous vertical stiffness model is put forward, using a continuous vertical stiffness model by considering a cable-stayed bridge deck as a beam–column system on an elastic foundation [13]. Accordingly, investigations and improvements on the buckling performance of cable-stayed bridges can be carried out on the girders due to the large axial forces on sections near the tower [14].

In contrast, cable-stayed bridges with unique-shaped towers may require extra attention for the specific geometric configurations of the tower. Studies on a cable-stayed bridge with a spatial diamond-shaped tower focus on the buckling problems in both longitudinal and transverse directions, proposing possible buckling modes with respect to different tower columns and the girder [15,16]. Other investigations involve the inverted Y-shaped tower, the arch-shaped tower, and the shell-shaped tower consisting of several arches, which indicate that the stress concentrations at various tubular intersecting joints should be carefully treated [17,18,19]. The focus on nonlinear buckling in local areas becomes particularly crucial for unique cable-stayed bridges, offering enhanced insights compared to traditional cable-stayed bridges.

On the other hand, the local buckling issue may be affected by long-term effects, e.g., corrosion issues. For cable-stayed bridges, especially those with unique-shaped towers, the consideration of corrosion involves various sources of uncertainties due to complicated steel component designs. The probabilistic study is quite useful for obtaining the reliability index, among which the Monte Carlo simulation is the commonly used method for the accuracy with large sample sizes, albeit at higher computational costs [20,21]. Other reliability analysis methods are also put forward to improve the computational efficiency and redundancy in the reliability analysis of cable-stayed bridges [22]. Accordingly, it can be stated that cable corrosion has a significant impact on critical buckling loads, though it may not affect the buckling mode of the girder [23]. The corroded steel girder is also discussed with respect to different buckling modes on stiffeners and webs induced by various corrosion patterns, resulting in obvious decreases in load-carrying capacities [24,25]. A reliability analysis also indicates that corrosion increases the likelihood of local or even global buckling for steel bridges, and corresponding maintenance strategies are indispensable [26]. Hence, the utilization of high-strength weathering steel can be an option, which lowers the risk of buckling due to the improved corrosion resistance [27,28]; the specific design for structures can be further verified based on quantitative analysis of the structural reliability.

Concerning the buckling behavior and the reliability affected by steel corrosion, this study involves a case study of the Dongping River Bridge in Shanghai, China, which is a cable-stayed bridge with three inverted V-shaped towers. The rest of the paper is organized as follows. In Section 2, the finite element (FE) model is established with respect to the local areas that are sensitive to local buckling. The buckling analysis is conducted in Section 3 to obtain the buckling safety factors, and the parametric study is given concerning key factors of the bridge design. The reliability analysis is carried out in Section 4 for further consideration of the long-term corrosion of steel members, and the design optimization is discussed for this bridge. In Section 5, the conclusion is drawn.

## 2. Model Description

### 2.1. Bridge Design

The study is based on the Dongping River Bridge in Shanghai, China, which spans a combination of 50 m + 78 m + 50 m, i.e., a total length of 178m [29]. The main girder of the bridge consists of two steel boxes, with an overall width of 20.2 m. The sections near the towers are widened to 26.1 m. The depth of the girder is 2.5 m, and this is maintained consistent over the entire longitudinal direction. The deck plate of the box girder has a thickness of 20mm, while the bottom plate and the web have a thickness of 16 mm.

**Figure 1:**
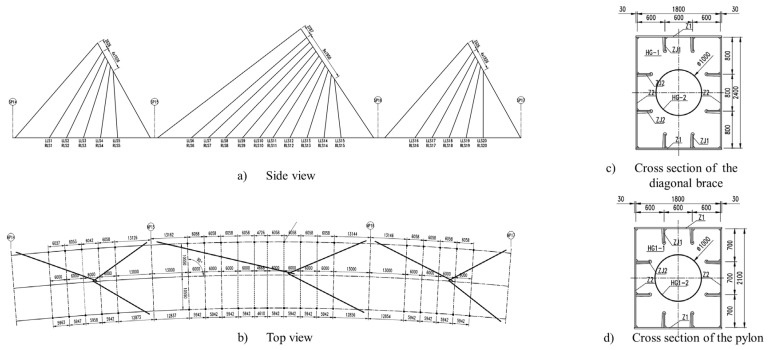
The details of the bridge structure (units: mm).

Each span of the bridge is supported by a spatial double cable-stayed system, with a total of 10 pairs of cables on the middle span, and 5 pairs on the side ones. The towers are designed in an inverted V-shape, inclined longitudinally and supported by diagonal braces. At first glance, the buckling can be a critical concern for the diagonal brace on the middle span, as it has a length that is considerably larger than other components. Also, the inclined tower is susceptible to buckling, as it experiences bending and compression forces due to the self-weight and cable tensions. The cross-section of the diagonal brace is 2.4 m × 1.8 m and the cross-section of the tower is 2.1 m × 1.8 m. There are eight stiffening ribs both in the diagonal brace and the tower column, and they all have a thickness of 25 mm. The distances between each of them are 0.6m in the horizontal direction and 0.8 m and 0.7 m in the vertical direction. The details of the bridge structure are shown in Figure 1.

### 2.2. FE Model

**Figure 2:**
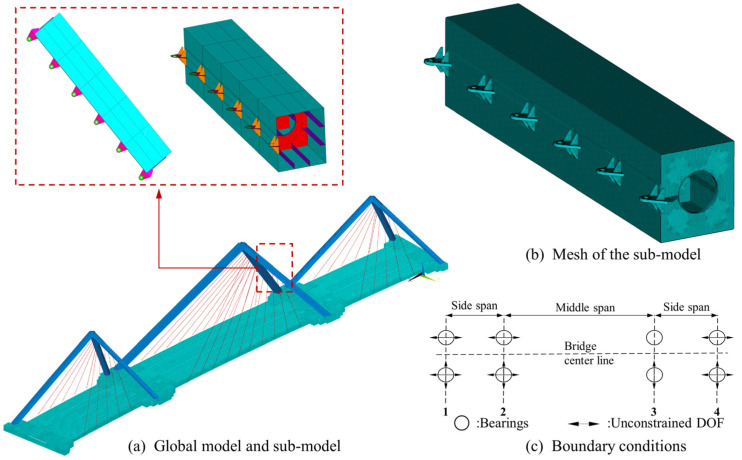
Full and local model of FEM: (**a**) Global model and sub-model; (**b**) Mesh of the sub-model; (**c**) Boundary conditions.

For the investigation on the buckling performance of the Dongping River Bridge, the FEA is conducted using ANSYS 19.0 finite element simulation software. A global model is first built, in which the main girder and towers are simulated using BEAM 188 elements, and LINK 10 elements are used for the cables. The global model is presented in Figure 2. With respect to the local buckling behavior of bridge components and members, a sub-model is also established. Based on the buckling analysis on the global model (as given in Section 3.1), it is implied that, due to the compression and bending forces induced by the cables and the inclined geometry, the inclined tower is the most vulnerable component for buckling. Hence, for the sake of the symmetrical geometry, the sub-model is established for part of the tower column. The sub-model consists of five box sections with six diaphragms, as shown in Figure 2. Shell elements are applied, namely the SHELL 181 element with four nodes and 6 DOFs for each, for the balance of computational efficiency and accuracy. The mesh size, as an important factor for the results in such studies [30], is also checked for the sensitivity of the results. Following the same analysis in Section 3.2, the element size of the sub-model is set to 0.02 m, as a similar result can be achieved when compared to that from a finer mesh. As a result, the sub-model consists of about 300,000 elements, as demonstrated in Figure 2b.

**Figure 3:**
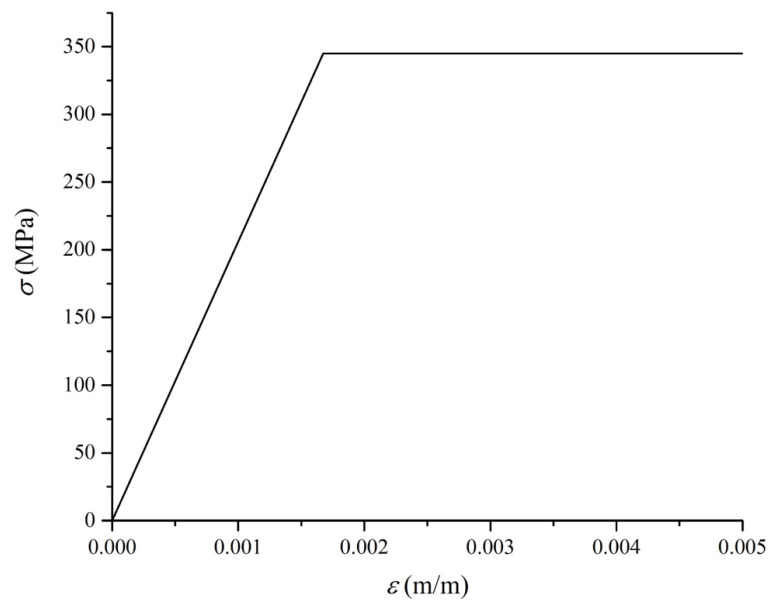
Ideal elastic–plastic constitutive model of Q345 steel.

The boundary conditions are consistent with the design, by constraining the degree of freedoms (DOFs) at the bearings, numbered as Bearing 1~4, as shown in Figure 2c. Specifically, all bearings on the north side constrain transverse displacements, and Bearing 3 further constrains the longitudinal direction on both sides. The material of the main girder and towers is Q345 steel, and the ideal elastic–plastic constitutive model is assumed, as shown in Figure 3. The material properties are provided in Table 1.

### 2.3. Loading Conditions

**Figure 4:**
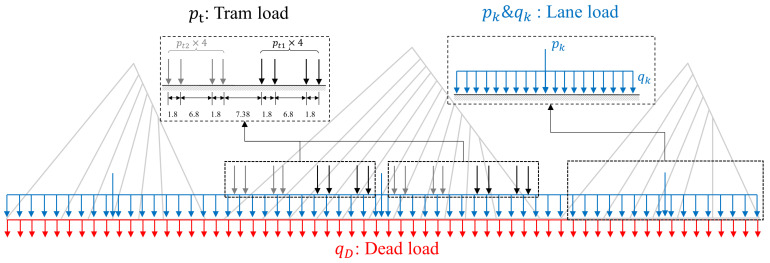
The dead load and live load on the bridge.

The loading conditions of the bridge are considered according to the design specifications. The cable forces are applied based on the values that were designed for its initial state. The dead load includes the self-weight of the structure and the secondary dead load of 65.5 kN/m, including the pavement layers, pedestrian barriers, medians, tram tracks, etc. As for the live load on the bridge, the lane load comprises a uniformly distributed load and concentrated forces on each span, following the Chinese design specifications [2]. In addition, as a potential action on the bridge, the tram load is also taken into account by assuming two trams on the middle span, each consisting of two carriages. The dead load and live load on the bridge are presented in Figure 4.

In the buckling analysis, the load factor can be used to amplify the loads on the bridge, until it reaches the critical value when the component buckles, namely the buckling safety factor. Two types of load case are considered: (a) Case 1: only the live load is amplified; (b) Case 2: both the live load and dead load are amplified, i.e.,

(1) $$\left\{\begin{array}{c} P_{case1}=\varphi \left(p_{t}+p_{k}+q_{k}\right)\\ P_{case2}=\varphi \left(p_{t}+p_{k}+q_{k}+q_{D}\right) \end{array}\right.$$

where φ is the load factor; $p_{t}$ is the tram load; $p_{k}$ and $q_{k}$ refer to the uniformly distributed part and concentrated part of the lane load, respectively; $q_{D}$ is the dead load. The amplification of the dead load is mainly for the comparison with the safety factors provided by standards and/or specifications, though the variations in self-weight and the secondary dead load are usually small in practical scenarios.

In addition, corrosion on steel members is a non-negligible issue, since the bridge is located at the mouth of the Yangtze River, exposed to saltwater and subject to a highly humid atmosphere. The buckling analysis of the bridge, especially when looking into the local buckling behavior of steel members, should be carried out concerning the thickness reduction under corrosion in the long term. In that case, the thickness of a certain steel member is regarded as a random variable following the normal distribution, and the standard value of the steel-plate thickness can be given by [31].

(2) $$t_{k}=t_{0k}-t_{\alpha }\left(1-\alpha \left(t\right)\right)$$

(3) $$\alpha \left(t\right)=\left\{1-\left[0.7\times {\left(t-t_{c}\right)}^{3}/1000\right]\times 0.04\times \alpha \times \xi \right\}$$

where $t_{k}$ is the standard value of the steel-plate thickness under a certain time of corrosion; $t_{0k}$ is the initial thickness; $t_{\alpha }$ is the reference corroded thickness; $t_{c}$ is the effective time of the anti-corrosion measure; α is the material parameter with respect to its corrosion resistance; and ξ is the environmental impact coefficient. Given the standard value, the mean and standard deviation of the thicknesses, i.e., $\mu_{t}$ and $\sigma_{t}$, can be given by

(4) $$\left\{\begin{array}{c} \mu_{t}=t_{k}/(1+1.645\delta_{t})\\ \sigma_{t}=\delta_{t}\mu_{t} \end{array}\right.$$

where $\delta_{t}$ is the coefficient of variance.

## 3. Buckling Analysis

In general, two types of analyses are commonly carried out in relation to buckling, namely the eigenvalue buckling analysis and the nonlinear buckling analysis. The eigenvalue buckling analysis falls within the category of a linear analysis method that is unable to consider the geometric nonlinearities caused by the large deformation of cables and towers, as well as the nonlinearities in material properties. Therefore, the focus is mainly put on the nonlinear buckling analysis to accurately account for the nonlinear behaviors of the bridge.

### 3.1. Global Model

**Figure 5:**
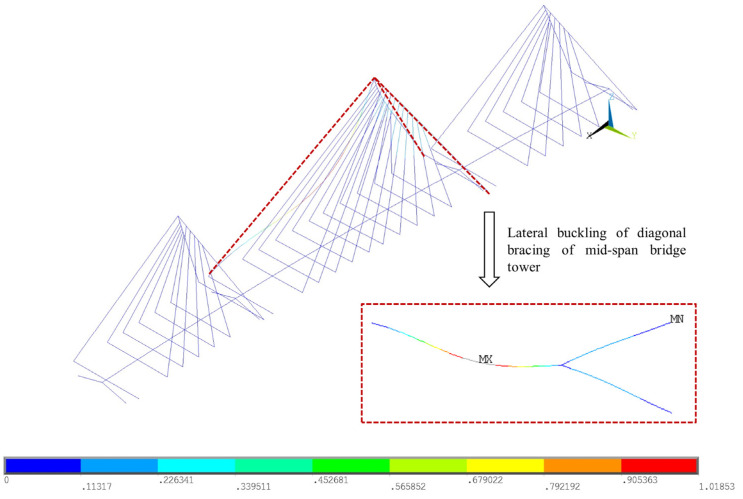
Eigenvalue buckling deformation.

Firstly, the analysis is performed on the global model to address the possible buckling mode. The eigenvalue buckling problem is solved by the generalized eigenvalue method. The results suggest that the buckling safety factor is 193.1 and the corresponding buckling mode is the bending of the diagonal brace on the middle span, as shown in Figure 5. However, as a solution to the first buckling problem, the result may not be conservative.

Subsequently, the nonlinear buckling analysis is conducted with consideration of the material properties and the large deformations. Based on the eigenvalue buckling analysis, the upper limit of the live load is determined. In addition, an initial deformation, representing geometric imperfections, is applied as 0.01 times the modal deformation. The buckling of the bridge is obtained through a load-incremental step-by-step solution based on the secant method. The load increment is based on the load factor α in Equation (1), and both Case 1 and Case 2, depicted in Section 2.3, are considered.

**Figure 6:**
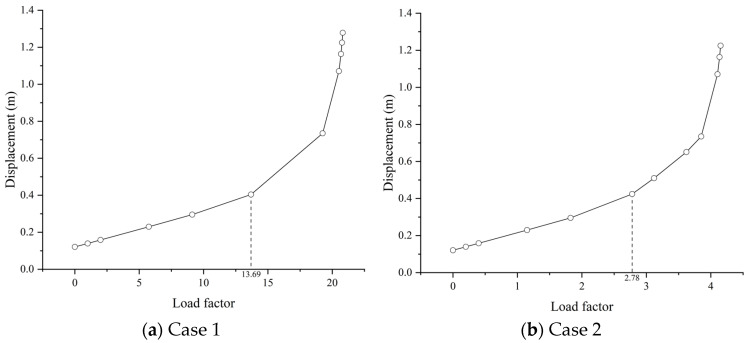
(**a**) The displacement of the buckling point on the tower column of Case 1; (**b**) The displacement of the buckling point on the tower column of Case 2.

The results imply that, in both cases, the buckling will initially occur on the inclined tower column on the middle span due to the bending moment caused by the cables, as well as the considerable axial force. The displacements of the buckling point on the tower column are presented for the load factor, as shown in Figure 6. Accordingly, the displacement is linearly changed with the increasing load factor in the beginning and then increases in a nonproportional way at a certain point, i.e., the buckling safety factor. The buckling safety factors for Case 1 and Case 2 are 13.69 and 2.78, respectively. Compared with the results in Figure 5, the difference can be attributed to the nonlinear buckling analysis that considers the elastoplastic constitutive model of the material. Moreover, the detailed model of the column geometry also introduces the stress concentration in local parts. Hence, it can be stated that the buckling safety factor given by eigenvalue analyses tends to be significantly overestimated, and the buckling mode is different as well.

By further checking the stresses on the structure, it can be noted that the Von Mises stress of the buckling point has reached the material yield strength when the load factor grows to the critical value, indicating that the divergence of displacement can be mainly attributed to the material nonlinearity. Nevertheless, the stresses on the global model are obtained simply by the beam theory, which are hardly accurate ones. Therefore, the stresses and behaviors of various elements on the tower column should be determined in a more detailed way to show the exact buckling of the structure.

### 3.2. Local Buckling

As the material nonlinearity is the main reason for buckling on the tower column, the stress on various details, especially the wall plates and the stiffening ribs, should be provided. In that case, a local buckling analysis is also carried out using the sub-model. The sub-model is established for the region of interest suggested by the above analysis on the global model, including the part of the tower column and six of the cable anchorages, as given in Section 2.2. Note that the effect from residual stress is not considered, since buckling can occur on a considerable range of the wall plate and ribs of the tower column, as presented below, while only a small part is affected by residual stresses near welded joints.

#### 3.2.1. Case 1

The structural responses with the increment of load factor in Case 1, namely *P_case_*_1_ in Equation (1), are first investigated for a more realistic depiction. Figure 6 presents the Von Mises stresses on the tower column under the dead load, in which the maximum value is 153 MPa at the corner of the box section. On this basis, the live loads are amplified by the load factor and applied to the FE model. The analysis is conducted in a step-wise way, and the load factor in each step is determined using the secant method.

**Figure 7:**
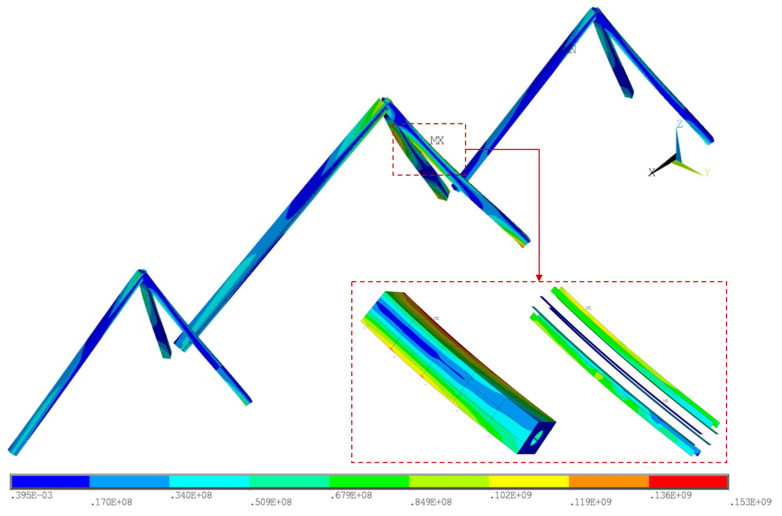
Von Mises stresses of the bridge tower under dead loads.

**Figure 8:**
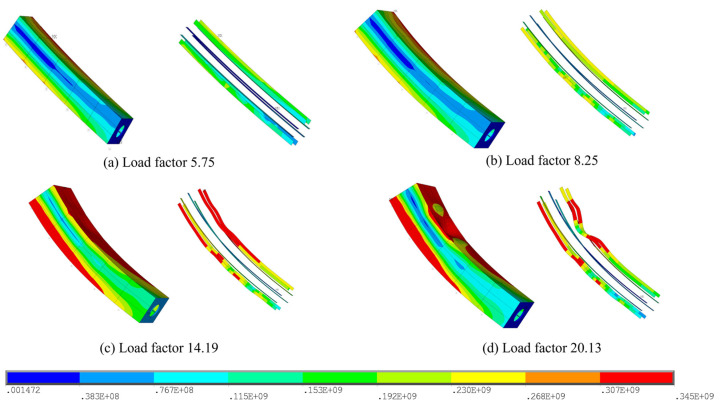
Von Mises stresses on the sub-model during the buckling (Case 1): (**a**) Load factor 5.75; (**b**) Load factor 8.25; (**c**) Load factor 14.19; (**d**) Load factor 20.13.

In Figure 7, the buckling progression on the tower column is illustrated, in which the Von Mises stresses on the wall plates and the stiffening ribs are given in an exploded view. The results show that, when the load factor is 8.25, the stresses of the wall plates at the corner of the box section approach the yield strength, i.e., the same position with the maximum stress under dead load, whilst the stresses of the stiffening ribs are still in the elastic stage, and thus the deformation of each element can be kept at a small level. Afterwards, the stiffening ribs near the cable anchorages and those on the opposite side will reach the yield strength when the load factor goes to 14.19, as shown in Figure 8. Note that the stiffening ribs near the cable anchorages turn to have a bending deformation, while those on the opposite side suffer from larger deformations because of buckling, and the connected wall plate presents the corresponding buckling behaviors, too.

**Figure 9:**
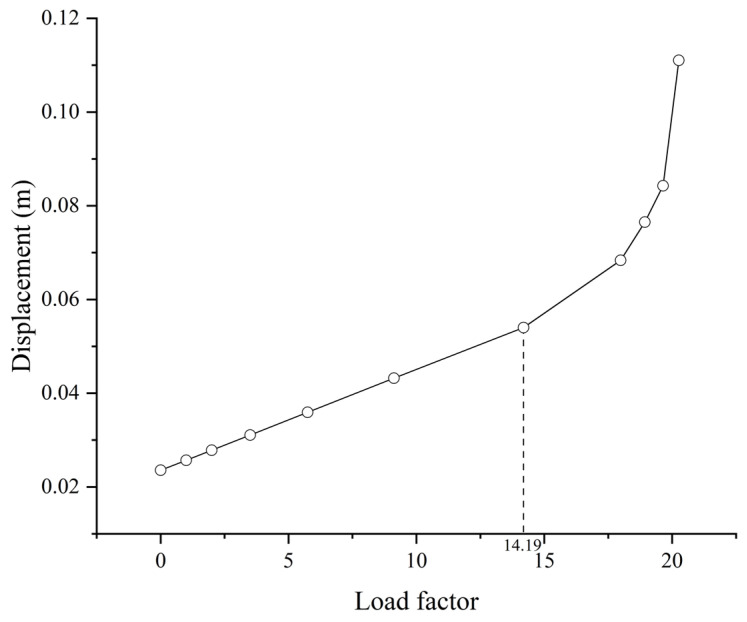
The displacement of the buckling point on the sub-model (Case 1).

Meanwhile, the node with the maximum displacement during the buckling in the sub-model can be determined, and its displacement is presented with respect to the load factor, as shown in Figure 9. It can be observed that the critical load factor is 14.19, which is slightly larger than the result obtained from the global model. The displacement is no longer proportional to the load factor after the critical value, and may reach about 0.112 m when the load factor is about 20.

#### 3.2.2. Case 2

In the same manner, the nonlinear buckling analysis is also conducted for Case 2, namely *P_case_*_2_ in Equation (1). The load factor is used to multiply both the dead load and the live load, with which the critical value can be comparable to the requirements in design specifications.

**Figure 10:**
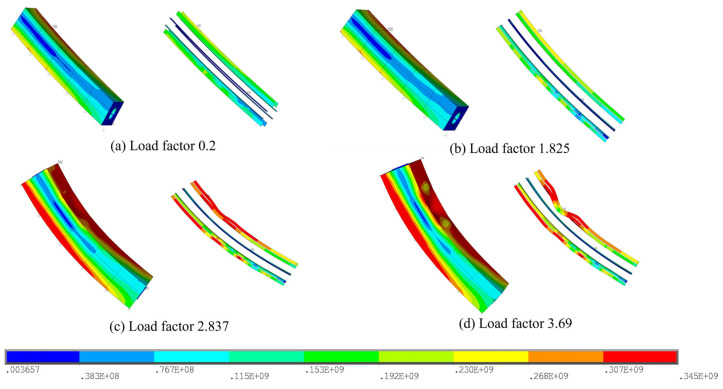
Von Mises stresses on the sub-model during the buckling (Case 2): (**a**) Load factor 0.2; (**b**) Load factor 1.825; (**c**) Load factor 2.837; (**d**) Load factor 3.69.

**Figure 11:**
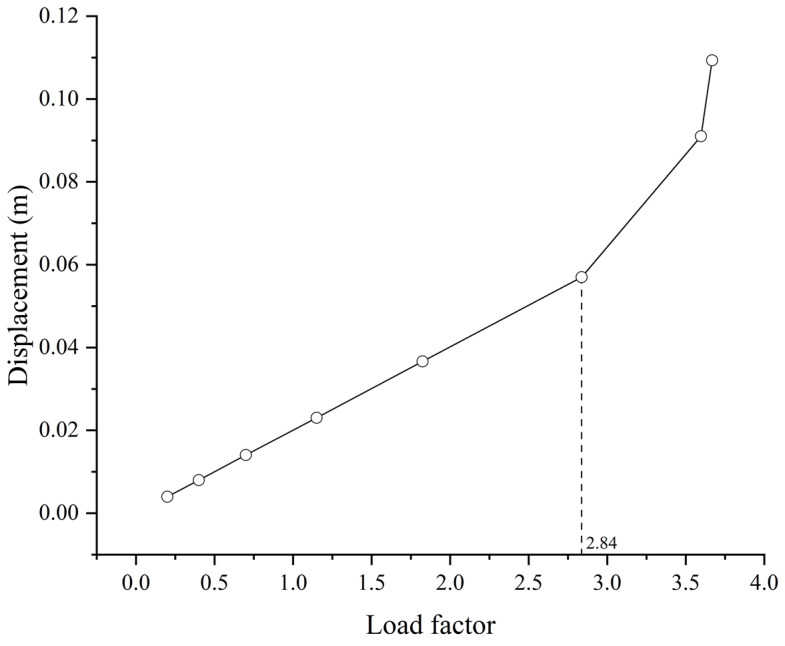
Displacement of the buckling point on the sub-model (Case 2).

Figure 10 illustrates the stresses of the sub-model during the buckling. The stress distributions and the deformations are basically the same as in Case 1. The obtained results indicate that the wall plates of the tower column will yield first at a load factor of 1.825, and then the stiffening ribs at a load factor of 2.84. Compared to Case 1, the critical value is significantly lower due to the substantial proportion of dead load. However, the deformation patterns of the wall plates and stiffening ribs remain similar. The displacement is also presented with respect to the load factor during the buckling, as shown in Figure 11.

Accordingly, it can be addressed that the potential buckling of the bridge can be attributed to the yield of steel materials locally. In particular, plastic deformation initiates on the wall plates before extending to the stiffening ribs of the tower column. Moreover, all cases exhibit the same buckling mode driven by cable tensions. As a result, the larger compressive forces and bending moments are imposed on the wall plates and stiffening ribs located on the side opposite the cable anchor plate.

### 3.3. Parametric Analysis

#### 3.3.1. Stiffening Ribs

Based on the analyses in Section 3.2, the buckling of stiffening ribs is the secondary effect following the yield of wall plates of the tower column. Hence, it provides ample room for the design optimization of stiffening ribs. In that case, the parametric analysis is performed concerning the thickness of stiffening ribs first.

**Figure 12:**
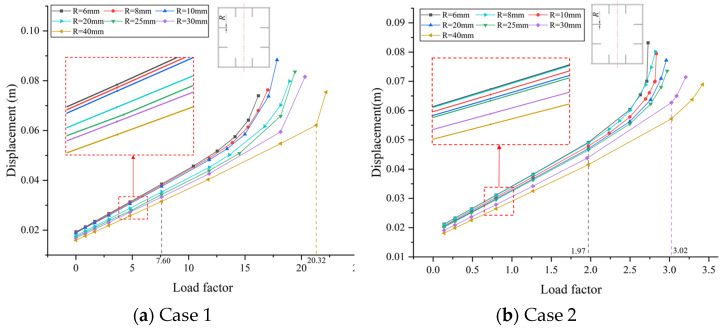
The effect of stiffening rib thickness with respect to load factor: (**a**) the load factor of Case 1; (**b**) the load factor of Case 2.

According to the bridge design, the thickness of stiffening ribs inside the box section of the tower column, *R*, is initially determined to be 25 mm. As part of a parametric analysis, this dimension is modified to 6 mm, 8 mm, 10 mm, 20 mm, 30 mm, and 40 mm. Subsequently, buckling analyses are conducted for both Case 1 and Case 2, and the resulting displacements of the buckling points on the sub-model are presented in relation to the load factor, as shown in Figure 12.

In both cases, it is evident that the displacement of the buckling point enlarges when the thickness of the stiffening rib is reduced, accompanied by a decrease in the buckling safety factor. Notably, when the rib thickness is set to 6mm, the resulting buckling safety factors are 7.60 and 1.97 for Case 1 and Case 2, respectively. Comparably, with *R* set to 40 mm, the corresponding buckling safety factors are 20.32 and 3.02. Nevertheless, it is noteworthy that even the smallest observed buckling safety factor of 1.97, identified at *R* = 6 mm, remains within the acceptable range defined by the design requirements. Though such thin stiffeners may not be suitable in actual projects, it demonstrates, as a parameter study, the limit regarding how far the thickness of the stiffening ribs can ideally be reduced.

On the other hand, a more rational approach involves modifying the width of the stiffening ribs, following the suggested thickness-to-width ratio in standards and codes. The thickness-to-width ratio is defined as:

(5) $$b_{0}/t=45\varepsilon_{k}$$

where $b_{0}$ is the plate width; t is the plate thickness; $\varepsilon_{k}$ is the correction factor for steel.

Therefore, the analyses on cases with different thicknesses are extended to include varying thicknesses, along with the adjusted widths for the stiffening ribs. In accordance with the above results, only the thicknesses smaller than the original design are considered. Here, cases for *R* equal to 6 mm, 8 mm, 10 mm, 12 mm, and 15 mm are investigated with the corresponding widths *D* of 216 mm, 288 mm, 360 mm, 432 mm, and 540 mm, respectively.

**Figure 13:**
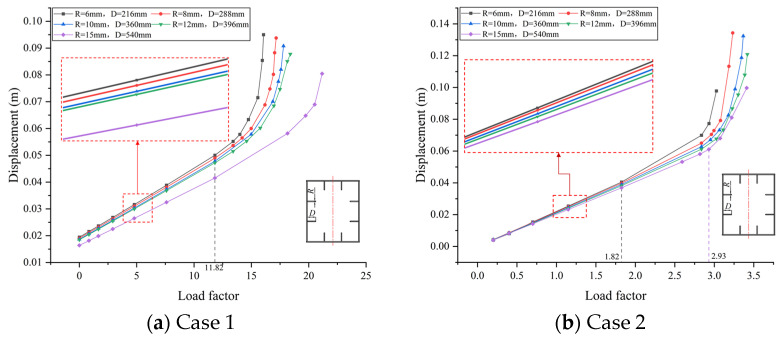
The effect of the width–thickness ratio of stiffening ribs with respect to load factor: (**a**) the load factor of Case 1; (**b**) the load factor of Case 2.

As a result, the load–displacement curves can be presented for both cases, as shown in Figure 13. In summary, it is evident that incorporating the recommended width–thickness ratio in the design significantly enhances the buckling resistance of the bridge. For each group of the design parameters, the buckling safety factor consistently remains around 11.82 for Case 1, and spans from 1.825 to 2.932 for Case 2. Hence, the smallest one is still enough for buckling safety, meaning that both the thickness and width of the stiffening rib can be optimized.

#### 3.3.2. Initial Cable Forces of Cables

Given the main effect of bending on the buckling of tower columns, the initial cable forces can be valuable for design optimization as well. Hence, the parametric analysis is performed concerning the normalized coefficient of initial cable forces, CCT, which is defined as follows:

(6) $$\mathrm{CCT}=T_{i}/T_{di}$$

where $T_{i}$ represents the adjusted initial tensile force of each cable in the FE model; and $T_{di}$ is the initial tensile force in the bridge design. Thus, the different values of CCT can be tested to demonstrate the effect of initial tensile forces of cables, and CCT = 0.7, 0.9, 1, 1.2, 1.4 is taken here.

**Figure 14:**
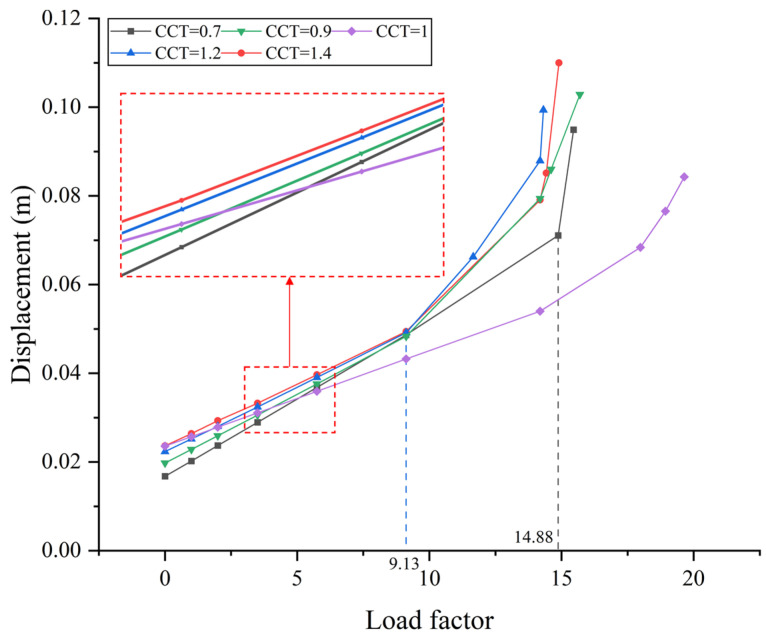
Effect of initial cable forces on load factor.

Using the same method described above, nonlinear buckling analyses are performed for different CCTs. To simplify the analysis without impacting the conclusions, the load factor was applied exclusively to live loads, referred to as Case 1 here. As a result, the load–displacement curve is given in Figure 14. It indicates that for CCT = 0.7, the buckling safety factor is approximately 14.88, representing a slight improvement compared to the original design (CCT = 1). Conversely, the buckling safety factor for CCT = 1.4 is around 9.13, indicating a less favorable scenario than the original design.

Hence, it suggests that the larger initial cable forces are unfavorable for the buckling concern of the bridge. On the other hand, advocating for a reduction in initial cable forces is not a practical recommendation, as the buckling safety factor can only rise by about 8.6%. In hindsight, the initial cable forces cannot play a dominant role, as they are roughly one order of magnitude smaller than those induced by the buckling loads. Given the sufficient buckling safety factor of the bridge, the improvement is quite limited and unnecessary.

## 4. Reliability Analysis

### 4.1. Monte Carlo Simulations

As the service time goes by, in consideration of the long-term implications related to the corrosion environment at the bridge location, an investigation is also imperative to address the buckling performance affected by the uncertain corrosion damages. Thus, it is of paramount importance to investigate the alteration in the load safety factor of steel structures caused by corrosion, ensuring long-term structural reliability. In this section, a reliability analysis is conducted using the Monte Carlo method, known for its accuracy and effectiveness in addressing high-order nonlinear problems.

Due to the possible initial damage and potential corrosion, the assumption is made that the thicknesses of stiffening ribs and the wall plates of the tower column follow normal distributions. According to Equation (2) and (3) in Section 2.3, the standard values of the thicknesses of stiffening ribs and wall plates after various in-service periods can be obtained. Then, numerous iterations are executed to provide the thicknesses in each case for the Monte Carlo simulation, following the mean and standard deviation given by Equation (4).

To distinguish from the parametric study, the design thickness of the rib is noted by $t_{1}$. As the parametric analysis implies the optimization, different values of 10 mm, 15 mm, 20 mm, and 25 mm are taken into account in the Monte Carlo simulation. To make a coupling analysis on parameters, the wall plate thickness, noted by $t_{2}$, is kept as the original design value of 30 mm, as the buckling behaviors may initiate at this location. Assuming that the effective action time of corrosion measures $t_{c}=5$, α=1.0 for steel structures with paint, and the environmental impact coefficient ξ=0.75. The coefficient of variance, $\delta_{t}$, is set as 0.04. Here, a 50-year service time of the bridge is considered with an interval of 10 years. The statistical parameters during the 50 years are presented in Table 2.

**Figure 15:**
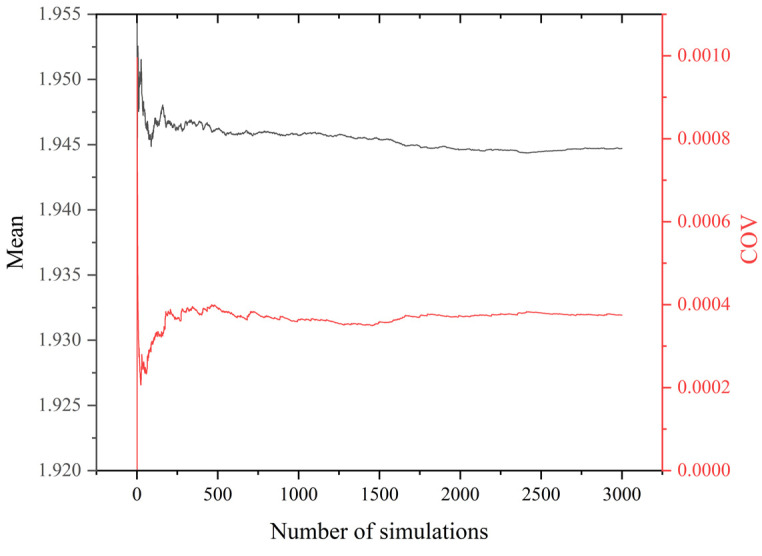
Buckling safety factors by Monte Carlo simulations ($t_{1}$ = 10 mm at 10 years).

Subsequently, the parameters can be used to update the FE model, and the Monte Carlo simulation is performed iteratively using ANSYS APDL finite element simulation software to achieve the random distributions of buckling safety factors. To determine the number of required cases, a total of 20,000 Monte Carlo simulations are conducted on the group of $t_{1}$ = 10 mm at 10 years. In terms of the buckling safety factor given in Figure 15, the mean value and the coefficient of variation (COV) stabilize after approximately 3000 simulations. For the sake of computational efficiency, therefore, each group of parameters in Table 2 is subjected to FE analysis using 3000 Monte Carlo simulations.

### 4.2. Results and Discussions

#### 4.2.1. Case 1

Firstly, the critical load factors in Case 1 are obtained for a more realistic depiction. The Monte Carlo simulations achieve critical load factors for varying rib thickness and for different service lives. It should be stated that the critical load factor is determined by the critical stress, rather than the displacement. Since the plastic deformation due to local yielding is the cause of buckling, it provides a valid insight and a conservative result for the reliability analysis by considering failure when the maximum stress on the tower column reaches the yield strength. Additionally, this approach significantly reduces the computational costs, as linear extrapolation can be applied.

**Figure 16:**
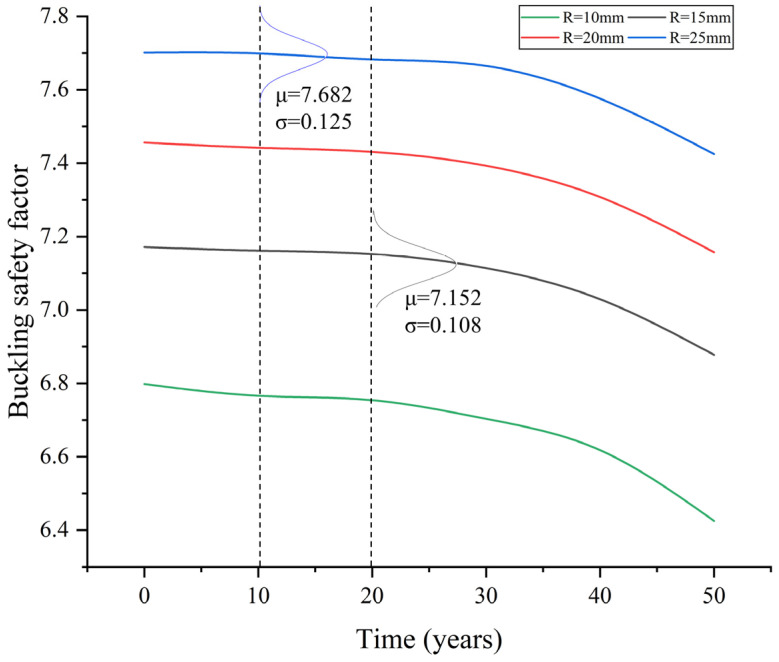
Critical load factors for different service periods for Case 1.

Accordingly, the critical load factors always follow a normal distribution. The mean values of the critical load factors are provided in Table 3 and Figure 16. The results indicate that, in the corrosive environment, the critical load factors decrease with increasing service time. A significant decline is observed after approximately 30 years of service, indicating extensive steel corrosion by that time. Comparing different rib thicknesses in design, thicker ribs are associated with higher critical load factors. For instance, when ribs are 25 mm thick, the critical load factor is approximately 7.5, whereas for ribs that are 10 mm thick, it is around 6.6.

Additionally, the Gaussian distributions of two cases are also depicted in Figure 16 as instances. It can be addressed that the standard deviation of the critical load factors is only about 0.1, and the corresponding coefficient of variance is about 0.015, primarily due to the small coefficient of variance of $t_{1}$ and $t_{2}$ in the assumption. As it is much smaller than the mean value, the discreteness of the results is negligible, validating the small number of Monte Carlo simulations conducted.

#### 4.2.2. Case 2

**Figure 17:**
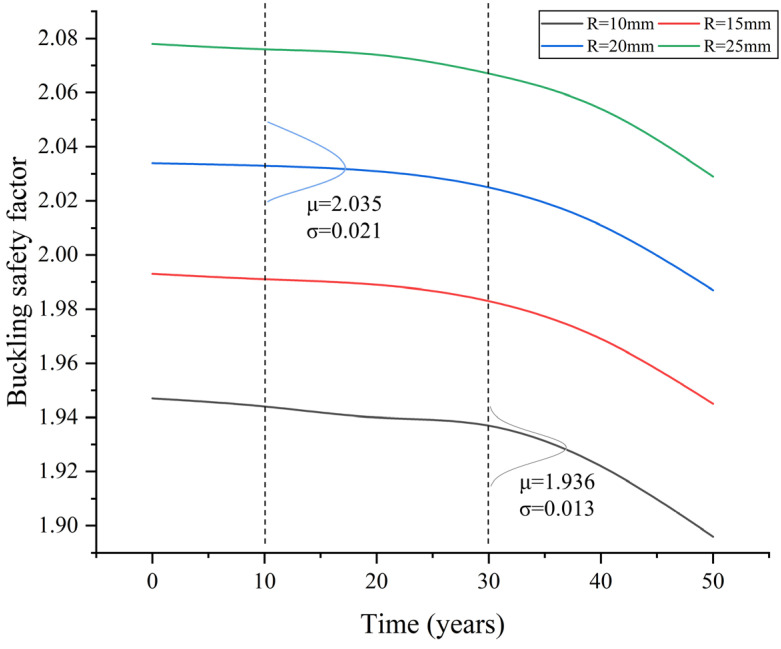
Buckling safety factor for different service periods for Case 2.

For Case 2, the buckling safety factor can be compared to the requirement in the design specifications, i.e., α≥1.75, so that the failure probability and the reliability index can be further obtained. Again, the normal distribution can be used to fit the results of buckling safety factors. For the simplification of the Monte Carlo simulation, it is assumed that the dead loads of the bridge and the live loads are kept consistent over a long period, and the stay-cable forces are assumed to remain constant as well. The mean values of the buckling safety factors are provided in Table 4 and Figure 17. Similarly, it can be observed that the clear decline of the buckling safety factors also begins after 30 years of service, and the coefficient of variance of the results is quite small. In that case, there is little if any chance that buckling safety factors decrease to be lower than 1.75.

Therefore, the buckling analysis is extended to 70 years after the bridge is put into service, and the buckling safety factor for the design of $t_{1}$= 10 mm is investigated, as the results are closer to the critical value. Table 5 presents the results of the reliability analysis. Accordingly, the failure probability is negligible for the first 50 years of service for the bridge, but experiences a significant escalation thereafter, yielding a reliability index of 0.12 after 70 years of service.

To sum up, the critical load factor of the bridge is always large enough when considering corrosion in the long term. Even for the reduced rib thickness of 10 mm, the bridge is unlikely to suffer from buckling failure in 50 years. For the current design, it is reasonable to assume the safety in terms of buckling. While considering an even longer period for buckling safety, the high-strength weathering steel with excellent corrosion resistance can be helpful for the design optimization of the tower column.

## 5. Conclusions

This study investigates the buckling behavior of a cable-stayed bridge with unique-shaped towers, i.e., the Dongping River Bridge in Shanghai, China. The bridge towers are subjected to compressive bending effects that raise the concern of buckling. Both global and local buckling behaviors are calculated by nonlinear buckling analysis. Furthermore, the reliability is further investigated with consideration of the corrosion-induced thickness reduction as a long-term concern. The following conclusions can be drawn from the study:

(1) The buckling of the bridge is primarily due to the plastic deformation of the tower column induced by large bending moments and axial forces. The wall plate of the column in the local part adjacent to the cable anchorage will yield initially, and then the stiffening rib in the vicinity follows to yield. The displacement will increase sharply afterwards, leading to the buckling failure of the column. The critical load factor is 14.19 when considering the amplification of live loads only, namely for Case 1, while it is 2.84 when considering the amplification of both dead loads and live loads, as in Case 2.
(2) Based on the parametric study with respect to the buckling safety of the bridge, the potential optimization of the design can be considered on the stiffening rib thickness and the width-to-thickness ratio. The thickness and the width of ribs can be reduced considerably in the design, while keeping a critical load factor of 1.825, still larger than the required one in the design specification. The initial cable forces can also be helpful for the effect of reducing the bending moments on the towers, yet are not recommended for the limited improvement, with possible influences on the geometrical configuration of the main girder.
(3) The reliability analysis was performed by using Monte Carlo simulations, considering the effects of corrosion on the steel components, including the stiffening ribs and wall plates. In addition, anti-corrosion measures and maintenance strategies are recommended after 30 years, since the reliability index will decrease sharply afterwards. Even with the optimized design featuring reduced rib thickness, the failure probability remains negligible after 50 years of service life. For a stiffening rib thickness of 10 mm, the reliability index drops to 0.12 after 70 years.

## Figures and Tables

**Table 1:** Material properties of Q345 steel.

Elastic Modulus	Density	Yield Stress	Poisson’s Ratio
206 GPa	7850 kg/m^3^	345 MPa	0.3

**Table 2:** Statistical parameters for stiffening rib and wall-plate thickness during service life.

Time		10 Years	20 Years	30 Years	40 Years	50 Years
$t_{1}=10\;\mathrm{mm}$	$\mathrm{Average}\;(\mu_{t1}$)	9.3817	9.1498	9.2748	9.0869	8.7542
	$\mathrm{Deviation}\;(\sigma_{t1}$)	0.3752	0.3659	0.3709	0.3634	0.3501
$t_{1}=15\;\mathrm{mm}$	$\mathrm{Average}\;(\mu_{t1}$)	14.0645	14.0506	13.9661	13.7782	13.4455
	$\mathrm{Deviation}\;(\sigma_{t1}$)	0.5625	0.5620	0.5586	0.5511	0.5378
$t_{1}=20\;\mathrm{mm}$	$\mathrm{Average}\;(\mu_{t1}$)	18.7643	18.7419	18.6574	18.4695	18.1368
	$\mathrm{Deviation}\;(\sigma_{t1}$)	0.7505	0.7496	0.7462	0.7387	0.7254
$t_{1}=25\;\mathrm{mm}$	$\mathrm{Average}\;(\mu_{t1}$)	23.4479	23.4332	23.3488	23.1608	22.8281
	$\mathrm{Deviation}\;(\sigma_{t1}$)	0.9379	0.9373	0.9339	0.9264	0.9131
$t_{2}=30\;\mathrm{mm}$	$\mathrm{Average}\;(\mu_{t2}$)	28.1471	28.1245	28.0401	27.8521	27.5194
	$\mathrm{Deviation}\;(\sigma_{t2}$)	1.1258	1.1249	1.1216	1.1140	1.1007

**Table 3:** Mean value of buckling safety factors for different service periods for Case 1.

Time		10 Years	20 Years	30 Years	40 Years	50 Years
Rib Thickness	10 mm	6.766	6.754	6.703	6.618	6.425
	15 mm	7.161	7.152	7.114	7.029	6.877
	20 mm	7.442	7.430	7.392	7.307	7.157
	25 mm	7.698	7.682	7.665	7.575	7.424

**Table 4:** Mean value of buckling safety factors for different service periods for Case 2.

Time		10 Years	20 Years	30 Years	40 Years	50 Years
Rib Thickness	10 mm	1.944	1.940	1.937	1.922	1.896
	15 mm	1.991	1.989	1.983	1.969	1.945
	20 mm	2.033	2.031	2.025	2.011	1.987
	25 mm	2.076	2.074	2.067	2.054	2.029

**Table 5:** Reliability analysis on the bridge in 70 years of service ($t_{1}\;$ = 10 mm).

Time	10 Years	20 Years	30 Years	40 Years	50 Years	60 Years	70 Years
Average buckling safety factor (μ)	1.944	1.940	1.937	1.922	1.896	1.847	1.753
Standard deviation (σ)	0.0193	0.0189	0.0192	0.0190	0.0191	0.0221	0.0301
Failure probability	4.26 × 10^−24^	3.51 × 10^−24^	1.40 × 10^−22^	6.99 × 10^−20^	1.17 × 10^−14^	5.63 × 10^−6^	4.52 × 10^−1^
Reliability index β	Inf	Inf	Inf	Inf	7.63	4.39	0.12

## Data Availability

The original contributions presented in the study are included in the article; further inquiries can be directed to the corresponding authors.

## List of Symbols

$P_{case1}$	Load combinations for Case 1
$P_{case2}$	Load combinations for Case 2
φ	Load factor
$p_{t}$	Tram load
$p_{k}$	Distributed part of the lane load
$q_{k}$	Concentrated part of the lane load
$q_{D}$	Dead load
$t_{k}$	The standard value of the steel-plate thickness under corrosion
$t_{0k}$	The initial value of the steel-plate thickness
$t_{\alpha }$	The reference corroded thickness
$t_{c}$	The effective time of the anti-corrosion measure
α	The material parameter with respect to corrosion resistance
ξ	The environmental impact coefficient
$\mu_{t}$	The standard mean value of the thickness
$\sigma_{t}$	The standard deviation value of the thickness
$\delta_{t}$	The coefficient of variance
*R*	The thickness of stiffening ribs
*D*	The width of stiffening ribs
$b_{0}$	The width of the wall plate of “Standard for the design of steel structures”
t	The thickness of the structure of “Standard for the design of steel structures”
$\varepsilon_{k}$	The correction factor for steel, which can be given by $\varepsilon_{k}=\sqrt{235/\sigma_{y}}$
CCT	The ratio coefficient of the cable force
$T_{i}$	The adjusted initial tensile force of each cable in the FE model
$T_{di}$	The initial tensile force in the bridge design
$t_{1}$	The design thickness of the stiffening rib
$t_{2}$	The original design of the wall plate
β	Reliability index

## References

[1] (2017). Standard for Design of Steel Structures.

[2] (2020). Specifications for Design of Highway Cable-Stayed Bridge.

[3] Huang B., Zhang W.F. (2020). Local-overall interactive buckling of high strength steel welded I-section columns under axial compression. Thin Walled Struct..

[4] Wang J., Di J., Zheng Y., Qin F., Su Y. (2024). Interactive buckling behaviour of Q420–Q960 steel welded thin-walled H-section long column. Thin Walled Struct..

[5] Wang W., Li X., Al-Azzani H. (2021). Experimental study on local buckling of high-strength Q960 steel columns at elevated temperatures. J. Constr. Steel Res..

[6] Kim S., Won D.H., Lee K., Kang Y.J. (2015). Structural stability of cable-stayed bridges. Int. J. Steel Struct..

[7] Madrazo-Aguirre F., Wadee M.A., Ruiz-Teran A.M. (2015). Non-linear stability of under-deck cable-stayed bridge decks. Int. J. Non-Linear Mech..

[8] Pedro J.J.O., Reis A.J. (2010). Nonlinear analysis of composite steel–concrete cable-stayed bridges. Eng. Struct..

[9] Chen C., Ma Y., Yan D., Li Z. (2020). Coupled nonlinear and time-dependent analysis for long span cable-stayed bridges. Struct. Infrastruct. Eng..

[10] Shu H.-S., Wang Y.-C. (2001). Stability Analysis of Box-Girder Cable-Stayed Bridges. J. Bridge Eng..

[11] Yoo H., Choi D.-H. (2008). New method of inelastic buckling analysis for steel frames. J. Constr. Steel Res..

[12] Yoo H., Na H.S., Choi E.S., Choi D.H. (2010). Stability Evaluation of Steel Girder Members in Long-Span Cable-Stayed Bridges by Member-Based Stability Concept. Int. J. Steel Struct..

[13] JPedro J.O., Reis A.J. (2016). Simplified assessment of cable-stayed bridges buckling stability. Eng. Struct..

[14] Xie X.L., Su H.L., Pang M.L. (2023). Mechanical Properties and Experimental Study of a New Laminated Girder Single Tower Cable-Stayed Bridge. Int. J. Steel Struct..

[15] Zheng X., Huang Q., Zheng Q.G., Li Z. (2022). Transverse buckling analysis of spatial diamond-shaped tower cable-stayed bridge based on energy approach. Struct. Eng. Mech..

[16] Zheng X., Huang Q., Zheng Q.G., Shen K.J. (2023). A Practical Longitudinal Buckling Analysis Method of Spatial Diamond-Shaped Tower Cable-Stayed Bridge. Int. J. Struct. Stab. Dyn..

[17] Yuan H.H., Chen R.L., Wu Q.X., Hu J., Tang Y. (2023). Experimental and numerical investigation of the mechanical behavior of a cable-stayed bridge with shell-shaped towers. Structures.

[18] Shi Z., Hu H., Li J. (2021). Axis optimisation of arch-shaped towers for high-speed railway cable-stayed bridges. Eng. Struct..

[19] Bruno D., Greco F., Blasi P.N., Bianchi E. (2013). A 3D nonlinear static analysis of long-span cable stayed bridges. Ann. Solid Struct. Mech..

[20] Filho J.O.F., da Silva L.S., Tankova T., Carvalho H. (2024). Influence of geometrical imperfections and residual stresses on the reliability of high strength steel welded I-section columns using Monte Carlo simulation. J. Constr. Steel Res..

[21] Song C., Kawai R. (2023). Monte Carlo and variance reduction methods for structural reliability analysis: A comprehensive review. Probabilistic Eng. Mech..

[22] VTruong H., Kim S.E. (2017). An efficient method of system reliability analysis of steel cable-stayed bridges. Adv. Eng. Softw..

[23] Lu W.A., He Z. (2022). Effect of cable corrosion on nonlinear elastic stability of girder in Cable-Stayed bridges with floating systems. Structures.

[24] Chiu C.-K., Liao I.H., Yamaguchi E., Lee Y.-C. (2023). Study on the simplified evaluation method of the remaining load-carrying capacity of a corroded steel I-girder end using FEA. J. Constr. Steel Res..

[25] Yamaguchi E., Akagi T., Tsuji H. (2014). Influence of corrosion on load-carrying capacities of steel I-section main-girder end and steel end cross-girder. Int. J. Steel Struct..

[26] Sharifi Y., Paik J.K. (2011). Ultimate strength reliability analysis of corroded steel-box girder bridges. Thin Walled Struct..

[27] Da G., Yang Z., Yang S., Chen Y., Li Z., Wang C., Xiao L., Zhang Z. (2024). Corrosion behavior of 700 MPa grade weathering steel with 4.0 wt% Ni and 5.0 wt% Cr in simulated marine atmospheric environment. Constr. Build. Mater..

[28] Kong Z., Yang B., Shi C., Yang F., Tao Q., Vasdravellis G., Vu Q.-V. (2024). Experimental and numerical investigation on the buckling behavior of corroded CST under axial compression. Structures.

[29] Bin H., Jun P., Xuefeng C., Xiaoyu Z. (2023). Study on the Stability of Special-Shaped Bridge Tower of Dongping River Bridge. Shanghai Highw..

[30] Piekarczuk A. (2023). Evaluation of limit curves double corrugated profiles of arched roofs. J. Constr. Steel Res..

[31] Wei L. (2010). Reliability Research on the Stability of Existing Reticulated Shell Structures.

